# Aggression, Impulsiveness, and Suicidal Tendencies Among Adolescents in the Iranian Region: A Descriptive‐Analytic Cross‐Sectional Study

**DOI:** 10.1002/hsr2.72075

**Published:** 2026-06-16

**Authors:** Fathola Mohamadian, Marzieh Rostamkhani, Yousef Veisani, Sattar Kikhavani, Sehat Aibod

**Affiliations:** ^1^ Department of Psychology, Psychosocial Injuries Research Center Ilam University of Medical Sciences Ilam Iran; ^2^ Department of Psychiatry, Faculty of Medicine Ilam University of Medical Sciences Ilam Iran; ^3^ Non‐communicable Diseases Research Center Ilam University of Medical Sciences Ilam Iran; ^4^ Department of Psychology Ilam University of Medical Sciences Ilam Iran

**Keywords:** adolescent, aggression, impulsivity, Iran, mental health, sex factors, suicide, suicide prevention

## Abstract

**Background and Aims:**

Aggression and impulsiveness are personality traits linked to adolescent mental health and suicidal tendencies. Although their associations with suicidality have been reported, evidence on their independent effects among Iranian adolescents, especially in high‐risk regions, is limited. This study examined the relationships between aggression, impulsiveness, and suicidal tendencies among adolescents in a region of Iran.

**Methods:**

This descriptive‐analytic cross‐sectional study was conducted on 200 male and female secondary school students selected through multistage random sampling from four high schools. Data were collected using the Multi‐Attitude Suicide Tendency questionnaire, Buss–Perry Aggression Questionnaire, Impulsiveness Scale, and a demographic checklist. Data were analyzed using independent t‐tests, Pearson correlation, one‐way ANOVA, and multiple linear regression in SPSS version 23.

**Results:**

Male adolescents reported significantly higher aggression scores than females (*p* = 0.03), while impulsiveness did not differ by gender (*p* > 0.05). Aggression differed significantly across academic grades (*p* = 0.02). Aggression showed a strong positive correlation with suicidal tendencies (*r* = 0.61, *p* < 0.001), whereas impulsiveness demonstrated a weak correlation (*r* = 0.18, *p* = 0.01). In regression analysis, aggression was a significant predictor of suicidal tendencies (*β* = 0.61, *p* < 0.001), explaining 36% of the variance (R² = 0.36), while impulsiveness was not a significant predictor. Academic performance was significantly associated with aggression and impulsiveness, whereas parental education and occupation were not.

**Conclusion:**

Aggression is the main independent predictor of suicidal tendencies among adolescents, particularly males, while impulsiveness has a limited role. Early identification of aggressive behaviors and school‐based prevention programs are therefore essential in high‐risk regions.

## 1 Introduction

Aggression is a critical personality trait that has profound implications for adolescent mental health and behavior. It can arise from interpersonal conflicts, neurological factors, and inadequate self‐regulation, and is often conceptualized as a maladaptive response to frustration and psychological stress [1, 2, 3]. Impulsiveness, closely related to aggression, refers to difficulties in inhibiting immediate reactions to sudden stimuli and can increase vulnerability to risky behaviors [4]. Together, these personality traits have been consistently identified as important predictors of suicidal ideation and behavior [5, 6], highlighting the interplay between aggression, impulsiveness, and self‐harm tendencies.

Globally, suicide remains one of the most pressing mental health challenges, with rates increasing disproportionately among adolescents and young people. The World Health Organization reported that suicide is the second leading cause of death for people aged 15 to 29, with thousands of adolescents dying every year; nearly 4600 deaths occur in the 10–14 age group, and for each adolescent who dies by suicide, there are between 100 and 200 attempts [7].

In Iran, although the overall suicide rate is lower than in some countries, regional analyses indicate substantial geographic variability, higher rates in certain areas. Statistical reports suggest an average annual suicide rate of 4.26 per 100,000 women and 10 per 100,000 men [8], and adolescent suicide remains a critical concern [9]. Risk factors identified in previous studies include age, gender, social support, academic pressure, family history, substance use, and mental health problems [10, 11, 12, 13, 14]. Notably, one region of Iran has experienced a persistent increase in suicide and suicide attempts among adolescents over the past three decades, with statistical data from 2016 showing that individuals aged 15–24 accounted for 53.3% of suicide attempts in this region [15].

Given the substantial impact of aggression and impulsiveness on suicidal tendencies, a focused examination of these psychological characteristics during adolescence is warranted [16, 17]. Previous research has demonstrated significant associations between aggression and suicidal ideation among adolescents, and evidence suggests that aggressive behaviors are prevalent in more than half of student populations [18]. Regression analyses have also identified life satisfaction, stressful life events, and family history of suicide as predictors of suicidal tendencies, particularly among adolescent girls.

Despite growing global and national concern regarding adolescent suicide, empirical studies that simultaneously examine aggression, impulsiveness, and suicidal tendencies within specific Iranian regions remain limited. Moreover, there is a lack of research employing integrated descriptive, correlational, and regression approaches to clarify the independent contribution of each psychological factor. In response to these gaps, the present study aims to examine the relationship between aggression, impulsiveness, and suicidal tendency among adolescents in selected regions of Iran.

The novelty of this study lies in its combined analytical approach and its focus on region‐specific adolescent populations, providing context‐sensitive evidence that can inform targeted mental health interventions in schools and community settings. By identifying high‐risk adolescents and clarifying the psychological mechanisms underlying suicidal behavior, the findings offer practical implications for prevention strategies and support evidence‐based policymaking in adolescent mental health.

## 2 Materials and Methods

### 2.1 Study Design

This study was a descriptive–analytical study with a correlational design, conducted to investigate the relationship between aggression, impulsiveness, and suicidal tendency among high school adolescents in a region of Iran. From an applied research perspective, the study is considered applied research.

### 2.2 Population and Sampling Method

The statistical population included all boys and girl's students enrolled in secondary schools in a region of Iran during the current academic year. In coordination with the Provincial Department of Education, the research team randomly selected four high schools (two for boys and two for girls). Within each selected school, students from different grade levels were chosen using simple random sampling. Eligibility criteria were defined prior to sampling. Inclusion criteria were: (1) being enrolled as a high school student in one of the selected schools during the study period; (2) age range of 15–18 years; (3) ability to read and understand the questionnaire items; and (4) willingness to participate in the study. Exclusion criteria included: (1) absence on the day of data collection; (2) incomplete questionnaires (more than 10% missing responses); and (3) withdrawal from participation at any stage of the study.

### 2.3 Sample Size

The sample size was determined using the standard formula for descriptive studies: *n* = (*z*^2 * *p* * (1 ‐ *p*))/*d*^2.

Assuming a 95% confidence level (*Z* = 1.96), an estimated proportion (*p*) of 0.5, and a margin of error of 0.07, the minimum required sample size was calculated as 196 students. To compensate for potential non‐response or incomplete questionnaires and to enhance statistical power, the final sample size was increased to 200 participants.

### 2.4 Instruments

This study used four instruments:

#### 2.4.1 Multi‐Attitude Suicide Tendency (MAST) Questionnaire

This 30‐item questionnaire includes four subscales: attraction to life, repulsion by life, attraction to death, and repulsion by death. Participants rated each item on a 5‐point Likert scale (1 to 5). Cronbach's alpha coefficients for the subscales ranged from 0.76 to 0.83 [19]. In the present study, only the total MAST score was used for analysis, and the subscales were not evaluated separately.

#### 2.4.2 Aggression Questionnaire (AQ) by Buss and Perry, 1992

The AQ consists of 29 items across four subscales: physical aggression, verbal aggression, anger, and hostility. Participants rate each item on a 5‐point Likert scale, and several items (such as items 9 and 16) are reverse‐scored. Previous studies have consistently demonstrated the questionnaire's validity and reliability, with Cronbach's alpha values ranging from 0.80 to 0.83. In an Iranian sample, Asadi Majreh and Akbari, the researchers reported an overall reliability coefficient of 0.96 [4].

#### 2.4.3 Impulsiveness Scale (IS)

Developed by Hershfield and colleagues [20] to assess impulsiveness in children and adolescents. The scale includes 19 true‐false items. A score of 1 is given for each correct match with the answer key, yielding a total score range of 0 to 19. The reported test–retest reliability of the scale was 0.854.

#### 2.4.4 Researcher‐Made Demographic Questionnaire

This form collected information on participants’ gender, grade level, parents’ education, and history of academic failure.

### 2.5 Data Collection Procedure

After obtaining the necessary approvals from the local Department of Education, a trained psychology graduate visited the selected schools. After explaining the purpose of the study, the researchers distributed the questionnaires in person. Participation was voluntary, and written informed consent was obtained from the students’ parents or legal guardians prior to data collection. Students also provided assent before completing the questionnaires.

### 2.6 Ethical Considerations

The study was conducted in accordance with ethical guidelines and the principles of the Declaration of Helsinki. Ethical approval was obtained from the Ethics Committee of Ilam University of Medical Sciences (Approval Code: IR.MEDILAM.REC.1398.047). Written informed consent was obtained from the parents or legal guardians of all participants, and student assent was obtained prior to participation. Confidentiality and anonymity were strictly maintained, and participants were informed of their right to withdraw from the study at any time without penalty.

### 2.7 Data Analysis

Data were analyzed using SPSS version 23 (IBM Corp., Armonk, NY, USA). Descriptive statistics, including frequency, percentage, mean, and standard deviation (SD), were used to summarize study variables. Inferential analyses included independent‐samples t‐tests to compare aggression and impulsiveness across gender, academic grade, and academic failure status, and Pearson correlation coefficients to assess associations among aggression, impulsiveness, and suicidal tendency. Multiple linear regression analysis was conducted to examine the predictive roles of aggression and impulsiveness in suicidal tendency, reporting B, β, R, R², adjusted R², F‐statistics, 95% confidence intervals (CI), and p‐values. One‐way ANOVA was used to compare mean scores across categories of academic GPA, parental education, and parental occupation. Effect sizes were calculated using η² for ANOVA and Cohen's d for t‐tests. All analyses were two‐tailed, with a significance level set at α = 0.05. Analytical and reporting procedures followed established methodological references [21, 22, 23] and complied with the SAMPL guidelines and related recommendations [24].

## 3 Results

Table 1 shows that boys exhibited higher aggression levels than girls, with 26% of boys versus 17% of girls in the “high” and “very high” categories. For impulsiveness, 20% of adolescents scored “very low” or “low,” and 38.5% scored “high” or “very high,” resulting in 80% of the sample in the moderate‐to‐high range. Suicidal tendency was moderate‐to‐high in 48.5% of participants, reflecting a substantial prevalence. These descriptive findings suggest noticeable gender differences and a notable presence of impulsiveness and suicidal tendencies among adolescents.

**Table 1:** Frequency distribution of aggression tendency, impulsiveness level, and suicidal ideation among adolescents (*n* = 200).

Indicator	Level	Girls/Boys	% Girls/Boys	Cumulative %
Aggression Tendency	Very low (G/B)	17/13	8.5/6.5	—
	Low (G/B)	30/23	15/11.5	—
	Moderate (G/B)	36/38	18/19	—
	High (G/B)	13/19	6.5/9.5	—
	Very high (G/B)	4/7	2/3.5	—
Impulsiveness	Very low	2	1	1
	Low	38	19	20
	Moderate	83	41.5	61.5
	High	59	29.5	91
	Very high	18	9	100
Suicidal Ideation	Very low	39	19.5	19.5
	Low	64	32	51.5
	Moderate	59	29.5	81
	High	28	14	95
	Very high	10	5	100

Abbreviations: B = Boys, G = Girls.

As shown in Table 2 presents descriptive statistics for impulsiveness and suicidal ideation by gender. The minimum impulsiveness score among adolescents was 0, and the maximum was 15. The mean impulsiveness was 8.80 (SD = 2.60). The girls had a mean of 8.65 (SD = 2.68), while the boys had a mean of 8.87 (SD = 2.62). The mean suicidal ideation score was 81.40 (SD = 14.60) for the total sample, 80.10 (SD = 15.30) for girls, and 82.71 (SD = 13.90) for boys, indicating a slightly higher suicidal tendency among boys. The observed range was 54–122.

**Table 2:** Descriptive statistics for impulsiveness and suicidal ideation by gender (n = 200).

Variable	Gender	Min	Max	Mean	SD	N
Impulsiveness	Girls	1	15	8.65	2.68	100
	Boys	0	15	8.87	2.62	100
	Total	0	15	8.80	2.60	200
Suicidal Ideation	Girls	54	114	80.10	15.30	100
	Boys	57	122	82.71	13.90	100
	Total	54	122	81.40	14.60	200

According to the findings presented in Table 3, boys exhibited significantly higher levels of aggression compared to girls (*t* = –2.260, *p* = 0.03, Cohen's *d* = 0.32), while no significant gender difference was observed in impulsiveness (*t* = –0.588, *p* = 0.56, *d* = 0.07). Furthermore, students in Grade 11 demonstrated higher aggression scores than those in Grade 10 (*t* = –2.404, *p* = 0.02, *d* = 0.35), although impulsiveness did not differ meaningfully between the two grades. Additionally, academic failure was not significantly associated with either aggression or impulsiveness; however, impulsiveness was marginally higher among students with a history of educational failure (*t* = 1.832, *p* = 0.07, *d* = 0.35).

**Table 3:** Independent *t*‐tests comparing aggression and impulsiveness across gender, grade, and academic failure (*n* = 200).

Variable	Group	*N*	Mean	SD	*t*	df	*p* value	Cohen's *d*
Aggression	Girls	100	76.21	19.77	–2.260	198	0.03*	0.32
	Boys	100	82.49	19.47				
Impulsiveness	Girls	100	8.65	2.68	–0.588	198	0.56	0.07
	Boys	100	8.87	2.62				
Aggression	Grade 10	100	76.02	19.54	–2.404	198	0.02*	0.35
	Grade 11	100	82.68	19.65				
Impulsiveness	Grade 10	100	8.78	2.38	0.107	198	0.92	0.01
	Grade 11	100	8.74	2.89				
Aggression	With Failure	100	85.71	19.62	1.247	198	0.21	0.29
	No Failure	100	78.87	19.81				
Impulsiveness	With Failure	100	10.00	2.11	1.832	198	0.07	0.35
	No Failure	100	8.67	2.66				

*Note:* * represents statistical significance at *p* < 0.05

The results in Table 4 indicate that aggression shows a positive and significant correlation with suicidal ideation (*r* = 0.606, *p* < 0.001), with this association being slightly stronger among boys (*r* = 0.613) than among girls (*r* = 0.593). Impulsiveness demonstrated a weak positive correlation with suicidal ideation (*r* = 0.182, *p* = 0.010), which was statistically significant in the total sample as well as among boys (*r* = 0.201, *p* = 0.044), but not statistically significant among girls (*p* = 0.111).

**Table 4:** Pearson's correlation between suicidal ideation, aggression, and impulsiveness.

Variable	Group	*r*	*p* value	*N*
Aggression	Total	0.606	< 0.001^a^	200
	Girls	0.593	< 0.001^a^	100
	Boys	0.613	< 0.001^a^	100
Impulsiveness	Total	0.182	0.010^a^	200
	Girls	0.160	0.111	100
	Boys	0.201	0.044^a^	100

a Significant at *p* < 0.05

A simultaneous entry method was used to construct the regression model using a simultaneous entry method and entered both predictor variables (aggression tendency and impulsiveness) into the model based on theoretical considerations and prior correlation analyses. This approach allowed them to assess the independent contribution of each variable to suicidal ideation [24].

Table 5 shows that the researchers used a simultaneous entry method to build the regression model. The model explained 36% of the variance in suicidal ideation (Adjusted R² = 0.361). Among the predictors, only aggression tendency significantly predicted suicidal ideation (*β* = 0.609, *p* < 0.001, 95% CI: 0.363–0.537), whereas impulsiveness did not significantly predict suicidal ideation (*β* = –0.011, *p* = 0.86, 95% CI: –0.717–0.599).

**Table 5:** Multiple Regression Analysis of Factors Affecting Suicidal Ideation among Adolescents in the Study Region.

Variable	Unstandardized coefficient (*B*)	Standard error	Standardized coefficient (β)	*t*‐value	*p* value	95% CI
Constant	46.212	3.863	—	11.963	< 0.001	38.6, 53.8
Aggression Tendency	0.450	0.044	0.609	10.200	< 0.001	0.363, 0.537
Impulsiveness	–0.059	0.331	–0.011	–0.179	0.86	–0.717, 0.599

*Note:* Model: *R* = 0.606, *R*² = 0.367, Adjusted *R*² = 0.361, *F* (2,197) = 57.20, *p* < 0.001

The results presented in Table 6 indicate that impulsiveness was highest among students with a GPA below 10 (14.00), followed by those with a GPA of 15–17 (9.50), and lowest in the 10–15 range (6.86), F (3,196) = 3.795, *p* = 0.01, η² = 0.056. This effect size suggests a small to moderate association between academic average and impulsiveness.

**Table 6:** Results of ANOVA for Impulsiveness and Aggression among Students Based on Demographic Variables.

Independent Variable	Dependent Variable	Groups	Mean	SD	*F*	*p* value	*η*²
Academic Average	Impulsiveness	< 10	14.00	—	3.795	0.01	0.056
		10–15	6.86	2.035			
		15–17	9.50	2.14			
		> 17	8.63	2.803			
Academic Average	Aggression	< 10	—	—	4.760	0.003	0.066
		10–15	64.14	15.48			
		15–17	85.63	15.99			
		> 17	78.20	20.13			
Father's Education	Aggression	Below Diploma	81.05	22.4	0.104	0.99	0.001
		Diploma	78.47	18.9			
		University Degree	79.67	20.25			
Mother's Education	Aggression	Below Diploma	83.08	18.4	0.841	0.47	0.009
		Diploma	78.44	21.11			
		University Degree	78.36	18.43			
Father's Occupation	Aggression	Self‐employed	79.48	21.05	1.901	0.11	0.019
		Unemployed	100.4	18.20			
		Employee	78.32	18.71			
		Homemaker	61.00	—			
		Cleric	96.00	—			
Mother's Occupation	Aggression	Self‐employed	80.92	20.49	0.380	0.82	0.004
		Unemployed	64.00	38.18			
		Employee	78.55	17.30			
		Homemaker	79.45	20.10			
		Cleric	86.00	14.14			
Father's Education	Impulsiveness	Below Diploma	9.26	2.24	1.526	0.21	0.015
		Diploma	9.11	2.69			
		University Degree	8.45	8.45			
Mother's Education	Impulsiveness	Below Diploma	9.22	18.4	1.963	0.12	0.020
		Diploma	8.84	2.47			
		University Degree	8.28	2.86			
Father's Occupation	Impulsiveness	Self‐employed	8.77	2.96	0.439	0.78	0.005
		Unemployed	10.00	3.54			
		Employee	8.73	2.37			
		Homemaker	7.00	—			
		Cleric	10.00	—			
Mother's Occupation	Impulsiveness	Self‐employed	8.67	2.19	0.880	0.48	0.009
		Unemployed	8.50	4.95			
		Employee	7.86	2.97			
		Homemaker	8.91	2.60			
		Cleric	7.50	3.54			

Aggression scores were highest among students with a GPA of 15–17 (85.63), F (3,196) = 4.760, *p* = 0.003, *η*² = 0.066, indicating a modest effect of academic performance on aggression levels.

No statistically significant differences were observed for parents’ education or occupation (*p* > 0.05). Overall, academic GPA demonstrated a statistically significant but modest association with impulsiveness and aggression, whereas parental demographic characteristics were not significantly related to these psychological variables.

## 4 Discussion

The primary aim of the present study was to examine the relationship among aggression, impulsiveness, and suicidal ideation in adolescents in a region of Iran. The findings showed that male adolescents, compared to females, experienced higher levels of aggression and impulsiveness. Moreover, male adolescents also reported higher levels of suicidal ideation than female adolescents. These findings are consistent with previous studies [18, 25] and are in line with results reported by other researchers [17, 26]. Biological, psychological, and social differences between girls and boys may help explain these patterns. Specifically, male adolescents often face stronger social expectations related to emotional suppression and masculinity norms, which may reduce emotional regulation and increase vulnerability to aggressive and impulsive behaviors, thereby elevating suicidal ideation.

Correlation analysis indicated a significant positive relationship between aggression and suicidal ideation, with this association being stronger among boys than girls. This finding is consistent with earlier studies [4, 27, 28], suggesting that aggression can be considered a robust psychological correlate and predictor of suicidal thoughts in adolescents.

Although the relationship between impulsiveness and suicidal ideation was also positive and significant, its magnitude was comparatively weaker. Similarly, this association was stronger among boys than girls. These findings suggest that impulsiveness may contribute to suicidal tendencies, particularly when combined with poor emotional regulation and ineffective coping strategies in response to stress.

Multivariate analysis further demonstrated that even after controlling for impulsiveness, the relationship between aggression and suicidal ideation remained significant. This result underscores the independent and dominant role of aggression in predicting suicidal ideation among adolescents.

Regarding demographic variables, findings showed that second‐grade students exhibited higher levels of aggression compared to first‐grade students. Additionally, adolescents with a history of academic failure or lower GPA tended to report higher aggression and impulsiveness. Although some of these differences did not reach statistical significance, they remain clinically and psychologically meaningful. Academic difficulties, repeated failure experiences, and diminished self‐esteem may contribute to heightened emotional distress, which can manifest as aggressive or impulsive behaviors.

Parental education was also associated with adolescent aggression and impulsiveness. Specifically, lower parental education levels were linked to higher levels of these traits. While these associations were not statistically significant, they suggest a potential trend, indicating that parents with lower educational attainment may have fewer opportunities to acquire effective parenting practices and communication skills [29].

Furthermore, the father's occupation, particularly unemployment, was associated with increased aggression and impulsiveness in adolescents. This finding is consistent with previous studies [18, 30]. Economic instability and occupational stress may increase family tension, thereby negatively influencing adolescents’ emotional and behavioral adjustment, especially in sociocultural contexts where fathers are traditionally viewed as primary economic providers.

Adolescents with employed or clerical mothers demonstrated lower impulsiveness compared to those with homemaker mothers. This difference may be explained by variations in role modeling, autonomy support, and patterns of parent–child interaction.

In addition, several psychological and neurobiological mechanisms may underlie the observed relationships. For example, higher aggression in male adolescents may be linked to elevated testosterone levels, differential stress reactivity, and a greater propensity for externalizing behaviors [31, 32]. Likewise, impulsiveness may intensify suicidal ideation by impairing judgment and increasing susceptibility to risky behaviors during periods of emotional distress [33]. Contextual factors, such as peer dynamics, school climate, and exposure to violence, may further strengthen these associations, particularly among adolescents facing academic challenges or limited parental support [34].

Overall, the present study highlights aggression and impulsiveness as key predictors of suicidal ideation, with their effects shaped by demographic and familial factors, including gender, academic status, and parental education or occupation. These findings have important theoretical and practical implications, emphasizing the need for early preventive interventions, emotional regulation training, school‐based mental health programs, and targeted support for high‐risk adolescent groups.

### 4.1 Limitations and Future Works

Readers should take several limitations of this study into account when interpreting the findings. First, the data were collected using self‐reported questionnaires, which may be subject to response bias and social desirability effects. Second, the cross‐sectional design limits the ability to establish causal relationships between aggression, impulsiveness, and suicidal ideation. Third, the study focused on a specific region of Iran, which may restrict the generalizability of the results to other cultural or geographical contexts. Fourth, we did not apply structured diagnostic interviews such as MINI or SCID; therefore, we could not formally rule out pre‐existing psychiatric disorders or dominant personality traits, which represents a limitation of this study. Fifth, although the Multi‐Attitude Suicide Tendency (MAST) scale comprises multiple subdomains (attraction to life, repulsion by life, attraction to death, and repulsion by death), we only analyzed the total suicidal ideation score in the present study, and we did not perform separate analyses for each subdomain. This limits a detailed understanding of specific dimensions of suicidal tendencies among adolescents.

Future research should employ longitudinal and multi‐method designs, including clinical interviews and behavioral assessments, to better capture the dynamics of aggression, impulsiveness, and suicidal tendencies. We recommend conducting comparative studies across different regions and cultures to enhance the external validity of the findings. Moreover, future works should explore the role of protective factors such as social support, resilience, and school‐based interventions in mitigating suicide risk among adolescents.

## 5 Conclusion

This study demonstrated a significant relationship between aggression, impulsiveness, and suicidal ideation among adolescents in a region of Iran, with male students exhibiting higher levels across all three variables. Notably, aggression emerged as a stronger predictor of suicidal ideation than impulsiveness, particularly among boys, underscoring the need for gender‐sensitive and behavior‐focused prevention strategies.

In addition to psychological characteristics, the findings indicated that contextual factors such as academic performance, grade level, and parental education and occupation are meaningfully associated with suicidal tendencies, suggesting that social, economic, and structural determinants also contribute to adolescent mental health vulnerability.

Overall, the results highlight the importance of implementing integrated, school‐based mental health interventions, strengthening parental awareness and education, and improving access to professional psychological services for adolescents. By addressing both individual psychological traits and broader contextual influences, preventive efforts may more effectively reduce self‐harming tendencies and suicidal ideation among youth.

## Author Contributions

**Fathola Mohamadian:** conceptualization, methodology, writing – original draft preparation, supervision. **Marzieh Rostamkhani:** investigation, writing – review and editing. **Yousef Veisani:** formal analysis, data curation, validation. **Sattar Kikhavani:** resources, visualization, writing – review and editing. **Sehat Aibod:** data curation, project administration, writing – review and editing.

## Funding

This research received no financial support from any funding agency, institution, or organization. The funding bodies had no role in the design of the study; collection, analysis, or interpretation of data; writing of the manuscript; or the decision to submit the article for publication.

## Ethics Statement

This study was approved by the Ethics Committee of Ilam University of Medical Sciences (Approval code: IR.MEDILAM.REC.1398.047) and was conducted in accordance with the ethical standards of the Declaration of Helsinki.

## Consent

Written informed consent was obtained from the parents or legal guardians of all participants, and student assent was obtained prior to participation, in accordance with the approval of the institutional ethics committee.

## Conflicts of Interest

The authors declare no conflicts of interest.

## Transparency Statement

The lead author, Sehat Aibod, affirms that this manuscript is an honest, accurate, and transparent account of the study being reported; that no important aspects of the study have been omitted; and that any discrepancies from the study as planned (and, if relevant, registered) have been explained.

## Data Availability

The datasets generated and/or analyzed during the current study are available from the corresponding author upon reasonable request. The data are not publicly available due to ethical and privacy restrictions.
